# Physiological and pathological clinical conditions and light scattering in brain

**DOI:** 10.1038/srep31354

**Published:** 2016-08-11

**Authors:** Tsuyoshi Kurata, Sachiko Iwata, Kennosuke Tsuda, Masahiro Kinoshita, Mamoru Saikusa, Naoko Hara, Motoki Oda, Etsuko Ohmae, Yuko Araki, Takashi Sugioka, Sachio Takashima, Osuke Iwata

**Affiliations:** 1 Department of Paediatrics and Child Health, Centre for Developmental and Cognitive Neuroscience, Kurume University School of Medicine, 67 Asahimachi, Kurume, Fukuoka, 830-0011, Japan; 2 Community Medical Support Institute, Saga University School of Medicine, 5-1-1 Nabeshima, Saga, 849-8501, Japan; 3 Division of Neonatology, Yokohama City University Medical Center, 4-57 Urafune, Minami-ku, Yokohama, Kanagawa, 232-0024, Japan; 4 Central Research Laboratory, Hamamatsu Photonics K.K., 5000 Hirakuchi, Hamakita-ku, Hamamatsu, Shizuoka, 434-8601, Japan; 5 Faculty of Informatics, Shizuoka University, 3-5-1 Johoku Naka-ku, Hamamatsu, Shizuoka, 432-8011, Japan; 6 Yanagawa Institute for Developmental Disabilities, International University of Health and Welfare, 218-1 Tano-machi Mitsuhashi-machi, Yanagawa, Fukuoka, 832-0813, Japan

## Abstract

MRI of preterm infants at term commonly reveals subtle brain lesions such as diffuse white matter injury, which are linked with later cognitive impairments. The timing and mechanism of such injury remains unclear. The reduced scattering coefficient of near-infrared light (μ_s_’) has been shown to correlate linearly with gestational age in neonates. To identify clinical variables associated with brain μ_s_’, 60 preterm and full-term infants were studied within 7 days of birth. Dependence of μ_s_’ obtained from the frontal head on clinical variables was assessed. In the univariate analysis, smaller μ_s_’ was associated with antenatal glucocorticoid, emergency Caesarean section, requirement for mechanical ventilation, smaller gestational age, smaller body sizes, low 1- and 5-minute Apgar scores, higher cord blood pH and PO_2_, and higher blood HCO_3_^−^ at the time of study. Multivariate analysis revealed that smaller gestational age, requirement for mechanical ventilation, and higher HCO_3_^−^ at the time of study were correlated with smaller μ_s_’. Brain μ_s_’ depended on variables associated with physiological maturation and pathological conditions of the brain. Further longitudinal studies may help identify pathological events and clinical conditions responsible for subtle brain injury and subsequent cognitive impairments following preterm birth.

The survival rate of preterm infants has increased remarkably in recent decades^1^,^2^. However, a considerable proportion of preterm infants develop cognitive impairments and experience educational disadvantage at school age, which persists at least into early adulthood^3^,^4^,^5^. Recent studies are addressing the brain injury responsible for cognitive impairment following preterm birth.

Dyet and colleagues reported that approximately 80% of infants born before 30 weeks gestational age developed subtle white matter lesions on term MRI^6^. These lesions were associated with neuro-developmental outcomes at 18 months corrected age. Furthermore, white matter injury in preterm infants was associated with cognitive and motor developmental quotients at 2 years old, even when findings were corrected for overt cerebral lesions detected by cranial ultrasound^7^. This suggests that subtle brain lesions visible on term MRI may be responsible for later cognitive impairments.

However, MRI is usually obtained only once in newborn infants, when their respiratory condition becomes stable enough for them to go into an MRI scanner. It remains unclear which clinical event or condition is responsible for subtle MRI lesions and later cognitive impairments, owing to the lack of studies that serially scan the brain of preterm infants in the acute phase. Development of non-invasive biomarkers for micro-structural brain changes may reveal the timing and mechanism of subtle brain injury in preterm infants.

Cranial ultrasound sonography is one such non-invasive tool that can be serially applied even in immature newborn infants. However, diagnostic utility of ultrasound sonography has been established mainly for macroscopic destructive lesions, such as intraventricular haemorrhage and periventricular leukomalacia^8^. Near-infrared spectroscopy (NIRS) is an alternative non-invasive probe that can be serially applied. Conventional, continuous-wave NIRS has been used to monitor the fraction of oxygenated and deoxygenated haemoglobin (Hb) concentration within a tissue^9^,^10^. More recently, time-resolved (TR) NIRS has been developed based on a time-correlated single-photon counting technique. Whereas continuous-wave NIRS estimates tissue oxygenation using both absorption and scattering information of near-infrared light, TR-NIRS gives reduced scattering coefficient (μ_s_’) and absorption coefficient (μ_a_) independently^11^,^12^,^13^, leading to several advantages in using TR-NIRS as a non-invasive biomarker of brain metabolism. For example, (i) direct monitoring of μ_a_ may allow more precise estimation of tissue oxygenation, (ii) the mean optical pathlength can be obtained from the temporal profile, and (iii) absolute concentrations of oxygenated and deoxygenated Hb can be estimated. Further, as the chief contributor to the light scattering of tissues is their organelle content^11^, μ_s_’ of brain tissue may reflect changes in micro-structural complexity caused by physiological maturation and pathological events. Indeed, Ijichi and colleagues reported that, in newborn infants between 30 and 42 weeks gestation, brain μ_s_’ obtained shortly after birth was linearly correlated with the gestational age^14^. However, it remains unclear whether (i) μ_s_’ reflects the maturation of the brain even when extremely preterm infants of younger than 28 weeks are included, and (ii) μ_s_’ is associated with micro-structural brain tissue changes following extrinsic clinical events, such as drug administration and hypoxic-ischaemic stress.

We conducted a prospective study to identify intrinsic and extrinsic clinical variables associated with μ_s_’ of near-infrared light obtained from the brain in newborn infants, ranging in gestational age from 24 to 41 weeks.

## Results

Temporal profiles from the frontal region were available for all 60 newborn infants (Table 1). However, for the right and left temporo-parietal regions and the posterior region, data were not obtained for 21, 21, and 22 subjects, respectively, because of insufficient signals from the brain (n = 18), poor probe contact mainly due to the lack of flat surfaces (n = 16), and/or the use of a cap device for nasal respiratory support (n = 4). There were subtle but significant intra-subject differences in μ_s_’ values between the wavelengths and head regions (Table 2). The value of μ_s_’ was higher for the wavelength of 761 nm than for 791 nm, whereas μ_s_’ for 791 nm was higher than for 836 nm (both p < 0.001, adjusted for head regions). μ_s_’ in the frontal and left temporo-parietal regions was lower than in the right temporo-parietal (both p < 0.001) and posterior (p = 0.001 and 0.002, respectively) regions, respectively (adjusted for wavelengths). There were marked linear correlations in μ_s_’ (i) between the three wavelengths (all p < 0.001), and (ii) between the four head regions (all p < 0.001, Supplemental Table 1).

Univariate analysis showed that a greater gestational age, body weight and height, head circumference (all p < 0.001), and Apgar scores at 1 and 5 minutes after birth (p < 0.05 and 0.01, respectively) were positively associated with greater μ_s_’ (Table 3A and Supplemental Table 2). Conversely, antenatal glucocorticoid, requirement for mechanical ventilation (both p < 0.001), emergency Caesarean section (p < 0.05), higher cord blood pH and PO_2_ (p < 0.05 and 0.01, respectively), and higher blood HCO_3_^−^ at the time of study (p < 0.05) were associated with smaller values of μ_s_’. Of these, body weight and height, requirement for mechanical ventilation (all p < 0.01), head circumference, antenatal glucocorticoid, and blood HCO_3_^−^ at the time of study (all p < 0.05) were still associated with μ_s_’ even after adjustment for gestational age. Gestational age, requirement for mechanical ventilation, and blood HCO_3_^−^ at the time of study were assessed within the multivariate model by forward selection; all these variables showed significant correlations with μ_s_’ (all p < 0.01, Table 3B).

## Discussion

Our data demonstrated that the property of light scattering within the newborn brain depends on both intrinsic and extrinsic factors, such as antenatal glucocorticoid, gestational age, body size, emergency delivery, Apgar scores, requirement for mechanical ventilation, and blood gas data at birth and at the time of study. Considering that these variables are associated with physiological maturation and pathological conditions, low μ_s_’ might reflect maturation of and damage to the brain tissue, and that serial μ_s_’ measurements at cot-side may help identify clinical conditions and events associated with the subtle brain injury and later cognitive impairments in preterm infants.

Cognitive impairments following very preterm birth remain a serious problem^3^,^4^,^5^, which have been attributed to non-destructive subtle brain injury as well as classical destructive brain injury^6^,^7^,^15^. Studies have highlighted that cognitive impairments are not specific to very-preterm infants. A large-scale cohort study demonstrated that the risk for cognitive impairments at school age was significantly higher for full-term infants who required resuscitation but did not need further neonatal care compared with their peers who did not need resuscitation^16^. Another observational study reported that late-preterm infants, who were born at 34 to 36 weeks gestation, were at increased risk for behavioural problems and lower intelligence quotients at the age of 6 compared with their full-term peers^17^. Considering that subtle brain injury was commonly observed even in late-preterm and full-term infants who did not experience apparent hypoxic-ischaemic events^18^, such injury might result from different mechanisms occurring at different times to classical hypoxic-ischaemic brain injury. However, it is difficult to delineate the mechanism and onset of clinical events responsible for subtle brain injury in these newborn infants, as serial MRI studies of the acute phases before and after clinical events are burdensome and unethical.

In this study, we used TR-NIRS to assess whether the property of near-infrared light scatter within brain tissue reflects micro-anatomical brain tissue structures in association with brain growth, maturation, and potential stressors. The optical penetration depth within tissue is wavelength dependent, and near-infrared light has much greater depth of penetration compared with light of other wavelengths^19^. Unlike μ_a_, μ_s_’ of near-infrared light is not affected by Hb concentration, and is likely to be determined by the structural complexity of the tissue^11^. Ijichi and colleagues first demonstrated that μ_s_’ is positively correlated with the gestational age of newborn infants ranging 30 to 42 weeks gestation when assessed shortly after birth^14^, suggesting that μ_s_’ might provide a non-invasive cot-side biomarker to monitor maturational changes in micro-anatomical brain structure.

Our findings suggested that μ_s_’ of near-infrared light obtained within the first week of life was linearly dependent on gestational age, even when the cohort was expanded to include extremely premature infants of up to 24 weeks gestation. In addition to gestational age, μ_s_’ was consistently positively correlated with the body weight, body height, and head circumference of the newborn infant at birth, the relationships of which were still observed even after adjustment for gestational age. Since a solution of the diffusion theory for a plane semi-infinite medium was applied for the estimation of μ_s_’, observations might be biased by the size of the cranium, because a smaller cranium has a greater curvature. However, in the current study, data were collected only when probe contact was satisfactory onto a flat surface of the head, suggesting that the difference in curvatures only minimally biased the measurement, if any.

In addition to intrinsic variables associated with the maturational status of newborn infants, extrinsic variables and pathological conditions, such as antenatal glucocorticoid, emergency Caesarean section, low Apgar scores, higher cord blood pH, requirement for mechanical ventilation, and higher blood HCO_3_^−^ at the time of study, were associated with smaller values of μ_s_’. Considering that these clinical variables depend on the maturational status and body sizes of newborn infants^20^,^21^,^22^, there remains a risk that potentially important independent variables are eliminated from multivariate models^23^. However, antenatal glucocorticoid, requirement for mechanical ventilation, and blood HCO_3_^−^ at the time of study were identified as independent variables of μ_s_’ even after the effect was adjusted for the influence of gestational age. In the final model, low μ_s_’ values were explained by younger gestational age, requirement for mechanical ventilation, and lower blood HCO_3_^−^ at the time of study.

Antenatal glucocorticoid has been used to accelerate lung maturation for preterm labour^24^. However, its adverse effects on brain growth and neuro-developmental outcomes have been noted^25^,^26^. Together with emergency Caesarean section, low Apgar scores, and a requirement for mechanical ventilation, these variables may reflect deleterious clinical conditions, which alter the micro-anatomical structure of brain.

Foetuses and neonates often encounter hypoxia, hypercarbia and subsequent acidosis^27^, where cerebral vessels are dilated to secure sufficient cerebral blood flow^28^. In the current study, in contrast to our estimation, lower pH, PO_2_ and HCO_3_^−^ (but not higher PCO_2_) were associated with high μ_s_’ values. A subsequent increase in cerebral volume and intracranial pressure may cause a subtle change in micro-anatomical structure of brain tissue and an increase in μ_s_’ values. This hypothesis may need to be tested by comparing blood gas and TR-NIRS data before and after a change of ventilator-setting.

Scattering of light in tissue depended on the wavelength used, however, μ_s_’ values obtained using wavelengths of 761, 791, and 836 nm differed by only up to 4%, and were tightly correlated between each other. Values of μ_s_’ were also linearly correlated between the four head regions. However, μ_s_’ obtained from the posterior and right temporo-parietal regions were higher compared with those obtained from the frontal and left temporo-parietal regions. Relatively higher μ_s_’ values posteriorly might be explained by the difference in gyral and micro-structural brain maturation, which remains further advanced in posterior regions than in anterior regions^29^.

It is more difficult to explain the right–left difference in μ_s_’ values. In foetuses, differences in the size of the lateral ventricle have been reported between the hemispheres^30^. In preterm infants, characteristic anatomical brain features, such as scaphocephaly and left-side dominant ventriculomegaly have been explained by the head position during intensive care, where newborn infants are predominantly cared for with their head facing the right side of the body axis^30^,^31^. Future studies need to assess whether similar right–left difference in μ_s_’ is observed even for newborn infants who are exclusively cared for with their head supine.

We were able to explore factors determining μ_s_’ in a relatively large number of newborn infants, across a range of gestational ages. This allowed us to assess the contribution of several newly identified variables, adjusting for the primary independent variable, gestational age. Several limitations of this study need to be highlighted when interpreting the findings. As previously described, the dependence of μ_s_’ on several important variables might not be properly assessed because of collinearity between clinical variables^23^. Findings from univariate analysis, as well as those from multivariate analysis, would be important for future investigations. Full-term infants in our study population had a variety of clinical backgrounds, rather than being healthy full-term infants. Although we excluded newborn infants who were likely to have moderate to severe brain malformation or destructive brain lesions, our current findings need to be confirmed in healthy infants. Our data suggested that low μ_s_’ values might represent immature or injured cerebral tissue. However, μ_s_’ might alter with macroscopic cranial alterations, such as enlargement of subarachnoid space or sulci, in addition to microscopic structural brain changes. Future studies need to clarify whether low μ_s_’ values represent a transient delay in maturation or permanent destruction of the brain, and whether macroscopic anatomical features of the cranium influence μ_s_’ values. An extensive study of the current cohort is underway to investigate quantitative magnetic-resonance biomarkers and neuro-developmental outcomes associated with regional μ_s_’ values, whereas studies in translational animal models are required for direct comparisons between μ_s_’ and histopathological findings.

Our current findings suggest that μ_s_’ obtained from the newborn brain is likely to reflect both physiological maturational status and the pathological condition of the brain. Small gestational age, antenatal glucocorticoid administration, low Apgar scores, and a need for mechanical ventilation might lead to maturational disorder or destruction of the brain, which, in turn, may alter micro-anatomical brain structure and abnormal μ_s_’ values. Further research could validate whether micro-anatomical differences in the newborn brain can be assessed serially and non-invasively at cot-side. Monitoring μ_s_’ changes before and after specific clinical events, such as septicaemia, malnutrition, cardio-pulmonary failure, and excessive stress, could identify the mechanisms and timing of subtle brain injury.

## Methods

This study was conducted in compliance with the Declaration of Helsinki under the approval of the Ethics Committee of Kurume University School of Medicine. Informed parental consent was obtained for each participating infant.

Values are shown as mean (standard deviation), unless otherwise specified.

### Study population

Between February 2011 and November 2012, 509 newborn infants were admitted to a tertiary neonatal intensive care centre (Kurume University Hospital, Kurume, Fukuoka, Japan). During the period, approximately 1 day per week was assigned for the study. All infants, (i) who were within the first week of birth and were available for the initial data collection on days assigned for the study, and, (ii) who underwent (or were planning) blood gas analysis for clinical purposes within 24 hours of the study, were recruited. To investigate independent variables associated with μ_s_’ in the brain without severe destructive brain lesions, newborn infants with grade II or III intraventricular haemorrhage, periventricular leukomalacia, hydrocephalus, and other major cerebral anomalies were not included in the study cohort. Subsequently, we enrolled 60 infants (preterm and full-term) of 24–40 weeks’ gestation (mean 31.9 [4.2] weeks gestation and 1517 [651] g at birth, Table 1). These newborn infants initially required intensive care due to preterm birth (n = 51), intrauterine growth restriction (n = 4), congenital heart disease (n = 3), jaundice (n = 1), and congenital duodenal stenosis (n = 1).

### TR-NIRS system

We used a TR-NIRS system (TRS-10, Hamamatsu Photonics K.K., Hamamatsu, Shizuoka, Japan), which uses three wavelength (761, 791, and 836 nm) light pulsers to generate light pulses with a pulse width of approximately 100 ps, a pulse rate of 5 MHz, and an average power of 30 μW. A time-correlated single-photon counting method was used to achieve a temporal profile of the brain, which was fitted to a solution of photon diffusion equation for a plane semi-infinite medium using a nonlinear least square fitting method to obtain μ_a_ and μ_s_’ for the three wavelengths^12^.

### Data collection

Serial data collection was performed within 7 days after birth and approximately every week thereafter until discharge home. However, for the current study, we only analysed data from the first assessment for each newborn infant. Temporal profile data were acquired when the patient was asleep or calmly awake and their clinical condition was stable. The TR-NIRS probe was first inserted into a rubber holder (inter-optode distance, 3 cm), and was applied to a relatively flat part of the head so that the surfaces of the light source and detector were in the same plane. Ten-second data acquisition was repeated five times for each region by repositioning the probe each time. This was repeated for frontal, left and right temporo-parietal, and occipital regions. To minimise technical bias, data were collected in the same order and within 10 minutes. For further analysis, the mean μ_s_’ value was calculated for each brain region. Data were not collected from specific regions, where probe contact was poor, or where signals from the brain were insufficient. The data quality was inspected retrospectively for their reproducibility and degree of fit to the photon diffusion equation.

### Clinical information

The clinical information was obtained from the patient’s record including (i) maternal and antenatal variables (parity, multiple pregnancy, antenatal glucocorticoid, intravenous tocolysis, peterm rupture of membrane, and elective/emergency Caesarean section), (ii) variables at birth and admission (sex, gestational age, Apgar scores at 1 and 5 minutes, cord blood gas data, need for mechanical ventilation at the time of admission (including nasal continuous positive airway pressure), body weight and height, and head circumference, and (iii) variables at the time of study (postnatal age, percent body-weight loss after birth, and blood gas data within 24 hours of TR-NIRS data collection).

### Data analysis

Body weight, height, and head circumference at birth were expressed as percentile scores according to the New Japanese Neonatal Anthropometric Charts for Gestational Age at Birth as markers for intrauterine growth^32^. To simplify analysis, we first assessed whether μ_s_’ values were similar and/or inter-correlated between different wavelengths and head regions. Generalised estimating equations were used to assess intra-individual differences/correlations of μ_s_’ values between 3 wavelengths (or 4 head regions) without the influence of head regions (or wavelengths). Patients’ identification numbers were used to define μ_s_’ measures from a same patient, whereas the wavelengths and head regions were used as fixed variables (SPSS-21, IBM, New York, NY). Based on robust inter-wavelength and inter-regional correlations in μ_s_’, values for 760 nm in the frontal region were used as representative data thereafter.

We assessed the dependence of μ_s_’ values on 14 selected (Table 3) and 18 additional (Supplemental Table 2) clinical variables using a simple regression analysis. Because of the known linear correlation between gestational age and μ_s_’^14^, results were presented with/without adjustment for gestational age. For these exploratory analyses, p-values were not corrected for multiple comparisons. Independent variables for the final model were assigned based on the results of univariate analysis, collinearity, and clinical relevance (gestational age used as a mandatory variable), and were determined by forward selection.

## Additional Information

**How to cite this article**: Kurata, T. *et al*. Physiological and pathological clinical conditions and light scattering in brain. *Sci. Rep.* **6**, 31354; doi: 10.1038/srep31354 (2016).

## Supplementary Material

Supplementary Information

## Figures and Tables

**Table 1:** Clinical background variables of the study population.

Variables	Mean	SD	Number	Percent
Clinical background variables				
Parity (multipara)			29	48.3
Multiple pregnancy			21	35.0
Antenatal glucocorticoid			35	58.3
Intravenous tocolysis			32	53.3
Premature rupture of the membrane			7	11.7
Caesarean section			38	63.3
Emergency Caesarena section			30	50.0
Variables at birth and admission				
Sex (male)			25	41.7
Gestational age (week)	32.0	4.2		
Apgar score				
1 min.	6.3	2.1		
5 min.	8.1	1.5		
Cord blood pH	7.34	0.07		
Mechanical ventilation			24	40.0
Body weight (g)	1517	651		
Head circumference (cm)	28.2	3.6		
Height (cm)	39.2	5.1		
Variables at the time of study				
Post-natal age (day)	3.3	3.3		
Corrected age (week)	32.5	4.2		
Body weight loss (%)	2.1	3.0		

Abbreviation: SD, standard deviation.

**Table 2:** Within-subject difference in the reduced scattering coefficient between head regions and wavelengths.

A: Difference between head regions							
Head region	Mean	SE	95% CI		P		
			Lower	Upper	vs. Left temporo-parietal	vs. Right temporo-parietal	vs. Posterior
Anterior	5.58	0.19	5.20	5.96	ns	<0.001	0.001
Left temporo-parietal	5.70	0.24	5.23	6.17	—	<0.001	ns
Right temporo-parietal	6.37	0.27	5.84	6.90	—	—	0.002
Posterior	6.37	0.24	5.90	6.83	—	—	—
**B: Difference between wavelengths**							
**Wavelength**	**Mean**	**SE**	**95% CI**		**P**		
			**Lower**	**Upper**	**vs. 791 nm**	**vs. 836 nm**	
761 nm	6.12	0.20	5.72	6.52	<0.001	<0.001	
791 nm	5.99	0.19	5.61	6.38	—	<0.001	
836 nm	5.90	0.19	5.53	6.26	—	—	

Values are shown as estimated marginal mean, SE and 95% CI adjusted for wavelengths (A) or head regions (B). Note that SE and 95% CI were given for inter-individual differences, whereas statistical analysis compared the intra-individual difference in the reduced scattering coefficient between head regions or wavelengths.

Abbreviations: SE, standard error. CI, confidence interval.

**Table 3:** Independent variables of the reduced scattering coefficient of near-infrared light.

	Crude regression coefficient				Adjusted for gestational age			
	B	95% CI		P	B	95% CI		p
		Lower	Upper			Lower	Upper	
**A: Univariate model**								
Maternal and antenatal variables								
Antenatal glucocorticoid	−1.636	−2.342	−0.93	<0.001	−0.923	−1.713	−0.134	0.023
Emergency Caesarean section	−0.942	−1.718	−0.165	0.018	−0.657	−1.321	0.007	0.052
Variables at birth and admission								
Gestational age (week)	0.21	0.131	0.29	<0.001	Not applicable			
Apgar score								
1 min.	0.29	0.109	0.471	0.002	0.146	−0.027	0.32	0.096
5 min.	0.363	0.095	0.631	0.009	0.166	−0.083	0.414	0.188
Mechanical ventilation	−1.777	−2.465	−1.088	<0.001	−1.096	−1.897	−0.296	0.008
Head circumference (cm)	0.26	0.17	0.349	<0.001	0.176	0.037	0.316	0.014
Cord blood gas analysis								
pH	−7.089	−12.492	−1.687	0.011	−2.816	−7.783	2.151	0.261
HCO_3_^−^ (mEq/L)	0.049	−0.036	0.135	0.254	0.03	−0.041	0.102	0.398
BE (mEq/L)	0.021	−0.069	0.112	0.639	0.043	−0.032	0.118	0.256
Variables at the time of study								
Post-natal age (day)	−0.068	−0.192	0.057	0.280	−0.024	−0.129	0.082	0.652
Blood gas analysis								
pH	2.543	−3.737	8.822	0.420	−2.516	−8.02	2.989	0.363
HCO_3_^−^ (mEq/L)	−0.165	−0.326	−0.003	0.046	−0.168	−0.297	−0.039	0.012
BE (mEq/L)	−0.071	−0.227	0.085	0.368	−0.158	−0.283	−0.033	0.014
**B: Final model**								
Gestational age (week)	0.147	0.045	0.249	0.005				
HCO_3_^−^ at the time of study (mEq/L)	−0.172	−0.293	−0.051	0.006				
Mechanical ventilation at admission	−1.136	−1.957	−0.314	0.008				

Abbreviations: SD, standard deviation. CI, confidence interval. BE, base excess.
