# Supplementary material for: Physiological and pathological clinical conditions and light scattering in brain
Source: Sci Rep. 2016 Aug 11;6:31354. doi: 10.1038/srep31354 (PMC4980768; doi:10.1038/srep31354)
Supplement: Supplementary Information [file srep31354-s1.pdf]

Online Supplemental Information

Physiological and pathological clinical conditions and light scattering in brain

Tsuyoshi Kurata[12], MD; Sachiko Iwata[1], MD, PhD; Kennosuke Tsuda[13], MD, PhD; Masahiro Kinoshita[1], MD; Mamoru Saikusa[1], MD; Naoko Hara[1], MD; Motoki Oda[4], MSc, PhD; Etsuko Ohmae[4], MSc; Yuko Araki[5], PhD; Takashi Sugioka[2], MD, PhD; Sachio Takashima[6], MD, PhD; Osuke Iwata[1], MD, PhD.

Affiliations

1. Department of Paediatrics and Child Health, Centre for Developmental and Cognitive Neuroscience, Kurume University School of Medicine, 67 Asahimachi, Kurume, Fukuoka, 830-0011 Japan.
2. Community Medical Support Institute, Saga University School of Medicine, 5-1-1 Nabeshima, Saga, 849-8501 Japan.
3. Division of Neonatology, Yokohama City University Medical Center, 4-57 Urafune, Minami-ku, Yokohama, Kanagawa, 232-0024 Japan.
4. Central Research Laboratory, Hamamatsu Photonics K.K., 5000 Hirakuchi, Hamakita-ku, Hamamatsu, Shizuoka, 434-8601 Japan.
5. Faculty of Informatics, Shizuoka University, 3-5-1 Johoku Naka-ku, Hamamatsu, Shizuoka, 432-8011 Japan.
6. Yanagawa Institute for Developmental Disabilities, International University of Health and Welfare, 284-2 Kamimiyanaga, Yanagawa, Fukuoka, 832-0058 Japan.

Correspondence to:
Dr Osuke Iwata
Centre for Cognitive & Developmental Neuroscience, Kurume University School of Medicine, 67 Asahimachi, Kurume, Fukuoka, 830-0011 Japan
E-mail: o.iwata@orbix.uk.net Tel: +81 942 31-7565 Fax: +81 942 38-1792

Online Supplemental Table 1:

Correlations of the reduced scattering coefficient between head regions and wavelengths

A: Correlations between head regions

Head regions		B			p
		Average	95% CI		
			Lower	Upper	
vs. Anterior	Left temporo-parietal	0.745	0.5	0.99	< 0.001
	Right temporo-parietal	0.652	0.399	0.906	< 0.001
	Posterior	0.527	0.33	0.724	< 0.001
vs. Left temporo-parietal	Right temporo-parietal	0.647	0.518	0.777	< 0.001
	Posterior	0.605	0.405	0.805	< 0.001
vs. Right temporo-parietal	Posterior	0.567	0.266	0.867	< 0.001

B: Correlations between wavelengths

Wavelenghts		B			p
		Average	95% CI		
			Lower	Upper	
vs. 761 nm	791 nm	1.03	1.01	1.06	< 0.001
	836 nm	1.08	1.05	1.10	< 0.001
vs. 791 nm	836 nm	1.04	1.02	1.06	< 0.001

Values are adjusted for three wavelengths (A) or four head regions (B).

Abbreviations: CI, confidence interval.

Online Supplemental Table 2:

Independent variables for the reduced scattering coefficient (all variables)

	Crude regression coefficient				Adjusted for gestational age			
	B	95% CI		p	B	95% CI		p
		Lower	Upper			Lower	Upper	
Maternal and antenatal variables								
Parity (multipara)	0.38	-0.43	1.19	0.357	0.30	1.00	1.00	0.372
Multiple pregnancy	0.35	-0.50	1.20	0.412	0.44	-0.26	1.14	0.215
Antenatal glucocorticoid	-1.64	-2.34	-0.93	<0.001	-0.92	-1.71	-0.13	0.023
Intravenous tocolysis	-0.73	-1.53	0.06	0.070	-0.24	-0.95	0.46	0.492
PROM	0.66	-0.60	1.91	0.301	0.53	-0.52	1.57	0.316
c/s	-0.25	-1.09	0.59	0.557	-0.31	1.00	1.00	0.382
Emergency c/s	-0.94	-1.72	-0.17	0.018	-0.66	-1.32	0.01	0.052
Variables at birth and admission								
Sex (male)	0.39	-0.43	1.21	0.348	0.35	-0.33	1.03	0.311
Gestational age (week)	0.21	0.13	0.29	<0.001	Not applicable			
Apgar score								
1 min.	0.29	0.11	0.47	0.002	0.15	-0.03	0.32	0.096
5 min.	0.36	0.10	0.63	0.009	0.17	-0.08	0.41	0.188
Mechanical ventilation	-1.78	-2.47	-1.09	<0.001	-1.10	-1.90	-0.30	0.008
Body weight (g)	0.00	0.00	0.00	<0.001	0.00	0.00	0.00	0.008
Percentile score	0.00	-0.01	0.02	0.921	0.01	-0.01	0.02	0.327
Head circumference (cm)	0.26	0.17	0.35	<0.001	0.18	0.04	0.32	0.014
Percentile score	0.00	-0.02	0.02	0.962	0.01	-0.01	0.02	0.328
Height (cm)	0.19	0.13	0.25	<0.001	0.14	0.04	0.23	0.005
Percentile score	-0.01	-0.02	0.01	0.453	0.01	-0.01	0.02	0.228
Cord blood gas analysis								
pH	-7.09	-12.49	-1.69	0.011	-2.82	-7.78	2.15	0.261
CO2 (mmHg)	0.02	-0.02	0.05	0.375	-0.01	-0.04	0.02	0.546
O2 (mmHg)	-0.04	-0.06	-0.01	0.007	-0.02	-0.04	0.01	0.141
HCO3- (mEq/L)	0.05	-0.04	0.14	0.254	0.03	-0.04	0.10	0.398
BE (mEq/L)	0.02	-0.07	0.11	0.639	0.04	-0.03	0.12	0.256
Variables at the time of study								
Post-natal age (day)	-0.07	-0.19	0.06	0.280	-0.02	-0.13	0.08	0.652
Body weight loss (%)	0.01	-0.13	0.14	0.926	-0.03	-0.14	0.08	0.589
Blood gas analysis								

Haemoglobin (g/dL)	0.10	-0.07	0.28	0.250	-0.05	-0.20	0.11	0.555
pH	2.54	-3.74	8.82	0.420	-2.52	-8.02	2.99	0.363
CO2 (mmHg)	-0.04	-0.09	0.01	0.083	-0.01	-0.06	0.03	0.559
O2 (mmHg)	-0.01	-0.04	0.01	0.305	0.00	-0.02	0.02	0.911
HCO3- (mEq/L)	-0.17	-0.33	0.00	0.046	-0.17	-0.30	-0.04	0.012
BE (mEq/L)	-0.07	-0.23	0.09	0.368	-0.16	-0.28	-0.03	0.014
Lactate (mEq/L)	0.12	-0.31	0.56	0.567	-0.17	-0.54	0.20	0.353

Abbreviations: SD, standard deveiation. CI, confidence interval. BE, base excess. c/s, Caesarean section. PROM, premature rupture of membranes. N/A, not applicable.

Online Supplemental Figure 1:
Data collection in a representative newborn infant.

A-B: Photograph of the TR-NIRS probe used in the study.

C: Photograph of a representative newborn infant during the study.

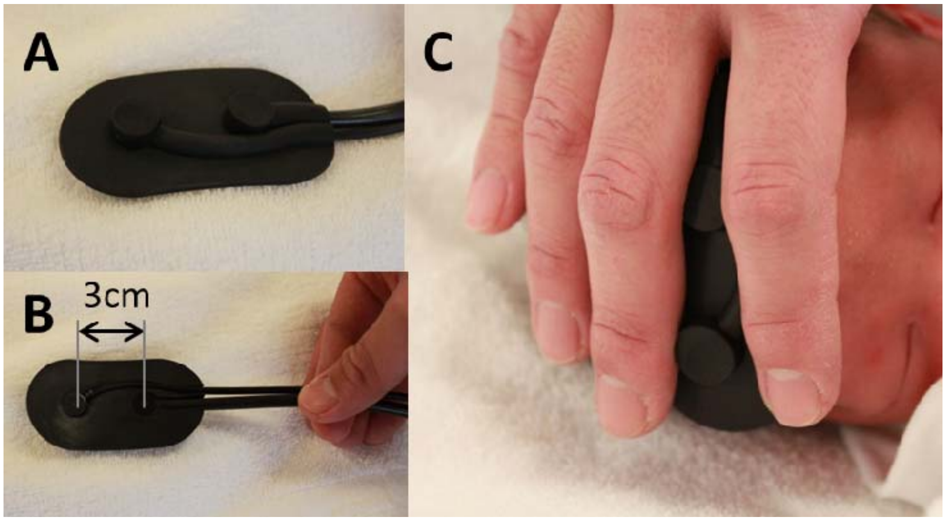

D: Cartoon depicting the head regions from where data were collected.

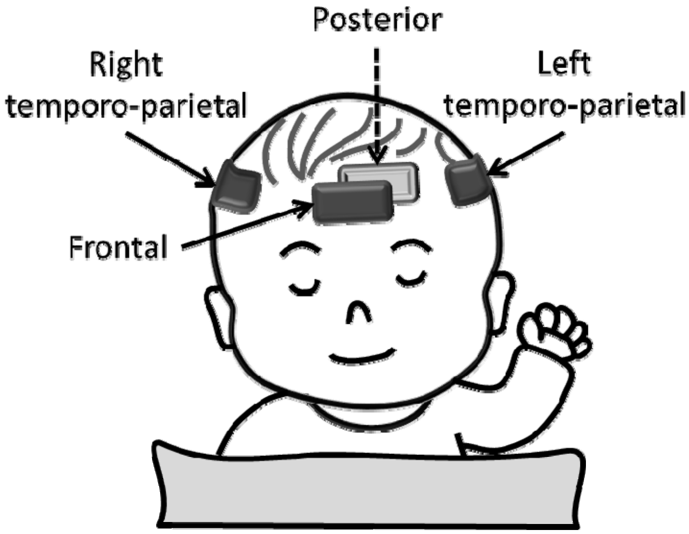

Online Supplemental Figure 2:

Dependence of the reduced scattering coefficient on clinical variables.

A-C: Binary variables (Data shown as mean + standard deviation)

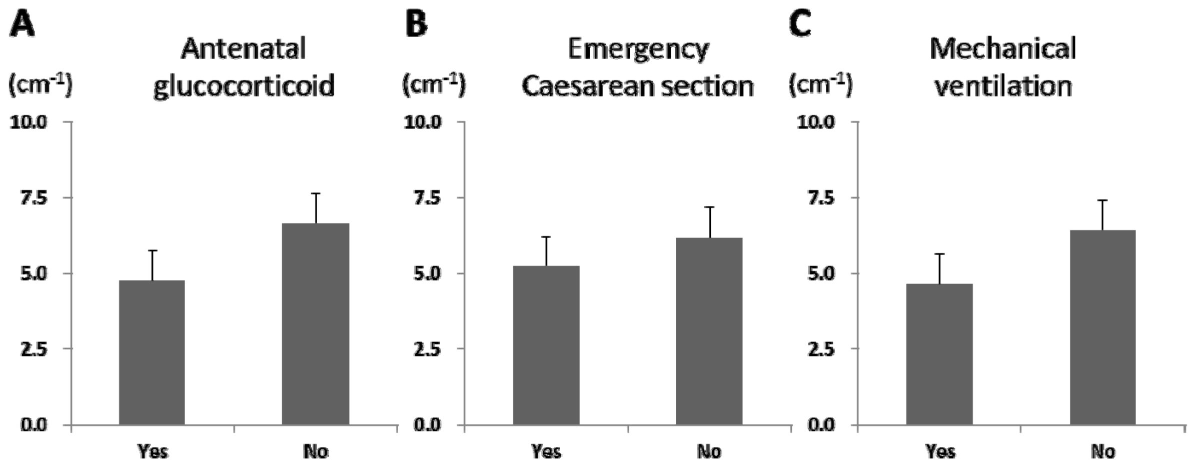

D-K: Correlations between independent variables and the reduced scattering coefficient.

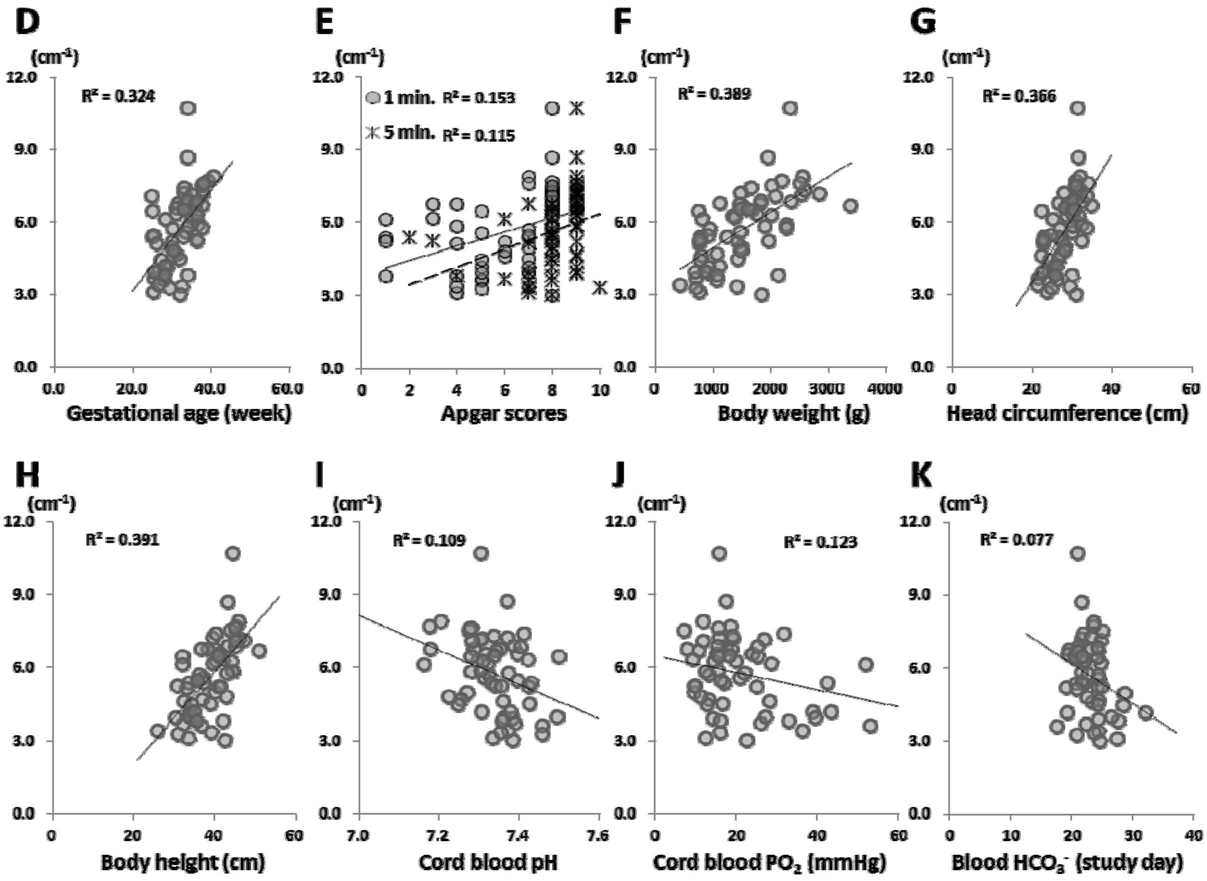

Regression lines are from simple linear regression analysis. R^2 are shown without adjustment for other variables.
